# Efficacy of Vancomycin and Meropenem in Central Nervous System Infections in Children and Adults: Current Update

**DOI:** 10.3390/antibiotics11020173

**Published:** 2022-01-28

**Authors:** Franziska Schneider, André Gessner, Nahed El-Najjar

**Affiliations:** Institute of Clinical Microbiology and Hygiene, University Hospital Regensburg, 93053 Regensburg, Germany; Franziska1.Schneider@ukr.de (F.S.); Andre.gessner@ukr.de (A.G.)

**Keywords:** meningitis, ventriculitis, central nervous infection, meropenem, vancomycin, pharmacokinetics, pharmacodynamics, target attainment

## Abstract

The current antimicrobial therapy of bacterial infections of the central nervous system (CNS) in adults and pediatric patients is faced with many pitfalls as the drugs have to reach necessary levels in serum and cross the blood-brain barrier. Furthermore, several studies report that different factors such as the structure of the antimicrobial agent, the severity of disease, or the degree of inflammation play a significant role. Despite the available attempts to establish pharmacokinetic (PK) modeling to improve the required dosing regimen for adults and pediatric patients, conclusive recommendations for the best therapeutic strategies are still lacking. For instance, bacterial meningitis, the most common CNS infections, and ventriculitis, a severe complication of meningitis, are still associated with 10% and 30% mortality, respectively. Several studies report on the use of vancomycin and meropenem to manage meningitis and ventriculitis; therefore, this review aims to shed light on the current knowledge about their use in adults and pediatric patients. Consequently, studies published from 2015 until mid-July 2021 are included, and data about the study population, levels of drugs in serum and cerebrospinal fluid (CSF), and measured PK data in serum and CSF are provided. The overall aim is to provide the readers a recent reference that summarizes the pitfalls and success of the current therapy and emphasizes the importance of performing more studies to improve the clinical outcome of the current therapeutical approach.

## 1. Introduction

Bacterial infections of the central nervous system (CNS) are a significant health concern affecting adults and pediatric patients [1,2]. Bacterial infections affecting different areas of the CNS include bacterial meningitis, brain abscesses, cranial and spinal epidural abscesses, as well as subdural empyema and ventriculitis, among others [3,4]. Bacterial meningitis, one of the most common CNS infections, is prevalent in low-income countries [5]. In a recent study on bacterial meningitis in adults, the in-hospital mortality for patients with community-acquired or healthcare-related meningitis was 10.6%, which increased to 14.8% at three months after discharge [6]. Ventriculitis, also accounted among the CNS infections, is a severe complication of meningitis and brain abscess that frequently occurs after neurosurgical procedures [2,7]. A recent study found that ventriculitis has an in-hospital mortality rate of 30% and leads to neurological sequelae in 60% of survivors, highlighting the severity of this type of infection [7]. A high cause for ventriculitis is the infection of an external ventricular drain (EVD) [8,9], commonly used to manage side effects associated with a severe brain injury such as hydrocephalus [10,11]. Unfortunately, although EVDs are necessary for handling side effects, their high risk of infection, even after implementing special control measures, still leads to a 10% prevalence of external cerebral ventricular drainage-associated ventriculitis in intensive care unit (ICU) patients [9]. All age groups can be affected by CNS infections and, if not timely diagnosed and treated immediately, can result in a high mortality rate [12,13]. Considering that quick intervention is necessary, empirical treatment with broad-spectrum antibiotics such as vancomycin is very common [12]. Vancomycin, in combination with β-lactams such as ceftazidime and meropenem, is used as an empiric treatment against healthcare-associated ventriculitis and meningitis [14].

Antibiotics used to treat CNS infections are given intravenously as a commonly used practice [14,15]. However, this strategy suffers from many challenges as a successful treatment in the case of CNS infections is not dependent on the serum concentration of the antibiotic but the achieved concentration in the cerebrospinal fluid (CSF) [16]. The antibiotics have to pass the blood-brain barrier (BBB) to reach the CSF. First, the penetration of an antibiotic into the CSF is dependent on the type and structure of the antibiotic itself. Additionally, some disease-specific factors influence antibiotics’ pharmacokinetics (PK) in CNS infections. Essential factors to consider in antibiotic dosage are meningeal inflammation, increased renal clearance, and drainage volume. For instance, many studies show that meningeal inflammation leads to higher penetration of antibiotics into the CSF [16,17]. Moreover, bacterial meningitis, which causes meningeal inflammation, was connected to a higher penetration rate than in other types of infection [18]. Therefore, the type of illness also impacts CSF drug penetration. Moreover, increased renal clearance, as often seen in ICU, affects antibiotic serum concentrations, leading to a different relation between dosing and CSF concentration [19,20]. Therefore, enhanced renal clearance must be considered to prevent underdosing in critically ill patients. Nevertheless, CNS infection also leads to increased CSF production that needs to be drained. As expected, several studies have shown that the drainage volume, which significantly impacts the PK of antibiotics in CNS infections, should be considered in the dosing of antibiotics in CNS infections to prevent underdosing in this vulnerable patient population [21,22,23]. Collectively, the intravenous dosage of antibiotics is strongly dependent on multiple factors and needs to be individualized for different patient groups.

To overcome the problems associated with the penetration of antibiotics through the BBB and the resulting variations in the antibiotics’ concentrations in the CSF, alternatives to intravenous administration such as intrathecal (direct injection into the cerebrospinal fluid) and intraventricular (direct injection into the cerebral ventricles) administration of antibiotics can be used [21,24,25,26,27]. However, more information needs to be gained on these alternatives because data on effective dosage are still limited [28].

An additional complication for CNS infection is adjusting the dosing regimens of drugs in children. Extrapolating the approved doses for adults to the lower body weight of children is not sufficient to ensure safe and effective use [15,29], and under- as well as overdosing of antibiotics in children is still an issue even with current dosing recommendations [30,31]. All of the aforementioned complications necessitate using other tools to predict the PK of drugs in different body compartments, such as serum and CSF, under different disease and age-related conditions.

A helpful tool is PK modeling, which creates a mathematical model that can predict the drug’s PK behavior [32]. PK models are usually divided into different compartments that represent the plasma (“central compartment”) and other tissues (“peripheral compartments”) [33]. The best possible mathematical description of the relationship between dosing parameters and the resulting concentrations is uncovered using patients’ data for validation. Important model parameters are the clearance (Cl) and the volume of distribution (VD) [32]. This latter one is calculated by dividing the amount of drug in the body by the drug’s plasma concentration. Consequently, to reach the same serum concentration, drugs with a high VD need to be given at higher concentrations than drugs with low VD [34]. Clearance, which corresponds to drug elimination, describes the volume of plasma that gets cleared of a drug per unit time [33]. Through statistical modeling, values for VD and Cl are generated to provide the best possible fit of the model and therefore lead to a minimal difference between predicted and measured concentration [32]. In CNS infections, multiple factors can influence the antibiotics’ PK [16,20,22]. Therefore, PK models can deliver valuable information about antibiotic dosing in different types of CNS infections. PK models for intravenously administered antibiotic concentrations in CNS infections usually contain a central, a peripheral, and a CSF compartment [20,35,36,37]. Extensive review about the generation of statistical modeling is out of the scope of this review and can be found elsewhere [32,33].

Vancomycin is recommended for empiric treatment of bacterial meningitis by the Infectious Diseases Society of America (IDSA, www.idsociety.org (accessed on 20 December 2021)) and the European Society of Clinical Microbiology and Infectious Diseases (ESCMIC, www.escmid.org (accessed on 20 December 2021)) while meropenem or other β-lactams are used as alternative or in combination therapy, based on local in vitro susceptibility pattern. Therefore, this review aims to highlight the current knowledge about the PK of meropenem and vancomycin in pediatric and adult patients suffering from meningitis and ventriculitis.

As a search strategy, the terms “meropenem” and “vancomycin” were combined with “CSF” or “cerebrospinal fluid”, and results from Pubmed and Google scholar from 2015 until 15 July 2021 were included. The PubMed search identified 125 and 69 papers for vancomycin and meropenem, respectively. The search in Google Scholar did not reveal additional publications. This review presents studies and case reports of meningitis and ventriculitis patients; studies on other types of infection were excluded. Included were only publications that contained PK data in the form of serum or CSF concentrations and/or described a PK model. The overall aim is to provide the readers with a recent reference that summarizes the pitfalls and success of the current therapy and improves the clinical outcome of the antibiotic therapy of patients affected by meningitis and ventriculitis.

## 2. Vancomycin

Vancomycin, a glycopeptide antibiotic, has a broad application area and is effective against most Gram-positive cocci and bacilli. It is predominantly used in methicillin-resistant *Staphylococcus aureus* (MRSA) infections and also against other Gram-positive β-lactam-resistant bacteria [38,39]. In combination with β-lactams such as ceftazidime and meropenem, vancomycin is used as an empiric treatment against ventriculitis and meningitis [14]. Due to its high molecular weight of ∼1450 Da and hydrophilicity, its penetration into the CSF when meninges are not inflamed is relatively poor [17,40]. Compared to meningitis, ventriculitis is associated with lesser meningeal inflammation [41], which means less CSF penetration. A study by Beach et al. found that penetration of vancomycin into the CSF is highly variable between patients with the same diagnosis and also between patients with different types of CNS infections. For instance, the maximum achieved CSF penetration (CSF to serum ratio) in adult meningitis patients was around 4-fold higher than in ventriculitis patients [42]. For vancomycin, the pharmacokinetic/pharmacodynamics (PK/PD) parameter that is most suitable for predicting outcomes in patients is the area under the curve (AUC) divided by the minimum inhibitory concentration (MIC) (AUC/MIC) [40]. Recommended total daily doses of vancomycin in healthcare-associated meningitis and ventriculitis are 30–60 mg/kg every 8 to 12 h in adults and 60 mg/kg every 6 h in children with normal renal and hepatic function. Alternatively, vancomycin can be administered as a 60 mg/kg/day continuous infusion. In adult patients who receive the intermittent bolus administration, it is recommended to maintain serum trough concentrations of 15–20 mg/L [14].

In the following, the main findings on the currently published studies on vancomycin use alone or in combination with meropenem in adults and pediatrics suffering from meningitis and ventriculitis are presented. Table 1 and Table 2 provide detailed information about the study population, sampling of blood and CSF, and measured PK data in serum and CSF.

### 2.1. Pediatrics

In the last six years, only three presented studies are reported on the use of vancomycin in pediatric patients (Table 1), all of which concern treatment of device-related ventriculitis. Hereby, two studies focus on intraventricular vancomycin treatment of infants.

Since only limited data are available on the use of intraventricular vancomycin in preterm infants, Parasuraman et al. conducted a retrospective study on the use of different doses ranging from 3 to 15 mg in ventriculitis affected infants born at less than 28 weeks gestation. The degree of ventricular dilatation was used as clinical guidance for the initial dosing. Intravenous vancomycin was used additionally at 15 mg/kg in the majority of the cases, and vancomycin was re-administered when CSF concentration was below 10 mg/L, which corresponds to 10 times the MIC levels for *coagulase-negative Staphylococci* of this center (1 mg/L). CSF drainage ranged from 5 to 10 mL/kg/day in all infants. The data demonstrated that larger intraventricular doses led to higher vancomycin concentrations in CSF that were maintained at sufficient levels for a longer period. At a dose of 3 mg, CSF levels were maintained above 20 mg/L for 18–24 h and declined under 10 mg/L at around 48 h after administration. There were also cases where CSF concentration declined faster, which is why the authors emphasize an individualized dosing regimen and recommend daily monitoring of CSF vancomycin levels. The authors did not observe adverse events that connect to the intraventricular administration route, and resolution of ventriculitis was accomplished in all patients in a median of 5.5 days (2–31 days) [25]. Consequently, Parasuraman et al. used the obtained data for a pilot population PK model. The best fit was provided by a one-compartment model, which revealed no appreciable transfer of vancomycin between plasma and CSF. In addition, CSF vancomycin levels were not significantly associated with the ventricular index and the CSF protein level. However, a larger data pool is needed to ascertain dosing strategies [43].

Matsunaga et al. aimed to determine optimal intraventricular administration of vancomycin for ventriculitis shunt infections in newborns. In this study, newborns were treated with the recommended doses for infants (10 or 20 mg vancomycin) and a lower dose of only 5 mg. In most cases, intravenous administration of 40.5 (±9.2) mg/kg/day was used concomitantly. Treatment with 20 and 10 mg led to CSF concentrations of over 20 mg/L after 78 h, which is above the recommended trough level of 5–10 mg/L in CSF for *Staphylococcus aureus* and *Staphylococcus epidermidis* infections. None of the treatment concentrations affected the auditory brain response. Nevertheless, two cases, treated with 10 and 20 mg vancomycin, relapsed. Interestingly, the study of Matsunaga et al. showed that also 5 mg vancomycin leads to a sufficiently maintained CSF concentration, reducing the risk of overdosing. Therefore, monitoring CSF concentrations should determine appropriate administration intervals [44].

In the retrospective study of Gibson et al., vancomycin serum trough levels were measured in patients with ventriculitis shunt infections caused by *coagulase-negative Staphylococci* (CoNS). Recommended serum trough concentrations of vancomycin in adults for this type of infection are 15–20 mg/L. Gibson et al. aimed to analyze whether this range of concentrations is also suitable for pediatric patients’ treatment success. Patients were treated with 15 mg/kg every 6 h. In 26.7% of patients, the antibiotic rifampin was used additionally. The median vancomycin trough level was 8.8 (5.4–27.7) mg/L, and in only 2 of 11 patients, the trough concentration was ≥15 mg/L. However, this did not negatively affect the treatment outcome. Since no recurrent infections occurred, Gibson et al. question the advantages of targeting vancomycin serum trough concentrations of 15–20 mg/L in children with CoNS shunt ventriculitis [45].

Collectively, it is evident that the current therapy of pediatric patients is not yet optimal, and (TDM) monitoring is needed to ensure achieving the required levels to eradicate the infection.

**Table 1:** Summary of vancomycins’ PK parameters in pediatrics affected with ventriculitis. *n* represents the number of participants. No data were determined about the CSF penetration.

Type of Infection	Study Design (*n*)	Dose	Route	Blood and CSF Sampling	Plasma (mg/L)	CSF (mg/L)	PK Model	Age	Treatment Outcome/ Remarks	Ref.
Ventriculostomy access device ventriculitis	Retrospective (8 cases, 7 patients)	3–15 mg IVT, redosage when CSF concentration < 10 mg/L, in 5 cases concomitant IV 15 mg/kg q24h (<29 weeks postmenstrual age) or q12h (29–35 weeks)	IVT (+IV)	Random CSF sampling (only in the presence of a clinical need for accessing the reservoir), 13 pre-dose CSF levels were available, no routine measuring of peak CSF, Source of CSF samples: intraventricular (Ommaya reservoir)	C_trough_ = 6.1 (<2–>100)	C_max_ (3 mg, 19 h) = 24.9 C_min_ (3 mg, 59 h) = 3.5 C_max_ (5 mg, 14 h) = 96.3 C_min_ (5 mg, 43 h) = 2.5 C_max_ (10 mg, 24 h) = 94 C_min_ (10 mg, 62 h) = 4.2 C_max_ (15 mg, 24 h) = 230.7 C_min_ (15 mg, 68 h) = 44.9	Yes [43]	GA: 25 + 4 weeks (23 + 6–27 +5 weeks)	Resolution in all patients in a median of 5.5 (2–31) days, No adverse events connected to IVT administration	[25]
Shunt ventriculitis	Retrospective (13 cases, 10 patients)	IVT 20 mg (6 cases)IVT 10 mg (2 cases)IVT 5 mg (5 cases)Concomitant IV in 8 cases (40.5 +/− 9.2 mg/kg/day)	IVT(+IV)	CSF samples 12 to 120 h following last IVT dose when CSF was absorbed to alleviate intracranial pressure, Source of CSF samples: intraventricular (Ommaya tube/ ventriculoperitoneal shunt)	C_trough_ = 12.3 ± 2.2	C(20 mg, 24 h) = 125.0 ± 30.1C(20 mg, 78 h) = 28.8 ± 0.8 (A)C(10 mg, 12 h) = 112.8 ± 17.8 (A)C(10 mg, 84 h) =23.2C(5 mg, 24 h) = 39.3 ± 22.9 C(5 mg, 72 h) = 16.5	No	GA: 34-week 5 day(±5 weeks 3 days)	Relapse in 2 cases (treated with 10 and 20 mg), Auditory brain response not affected by treatment	[44]
Shunt ventriculitis	Retrospective (30)	15 mg/kg q6h	IV	Serum samples 3 (2–5) days after initiation of treatment, only from 11 patients	8.8 (5.4–27.7)	ND	No	15.5 (1–192) months	No recurrent infections	[45]

Abbreviations: C_max_: maximum concentration, C_min_: minimum concentration, C_trough_: trough concentration, GA: gestational age, IV: intravenous, IVT: intraventricular, ND: no data, q12h: every 12 h, (A) not reported in study, calculated from individual patient data.

### 2.2. Adults

#### 2.2.1. Vancomycin in Ventriculitis

In all studies on the use of vancomycin in ventriculitis in adult patients (Table 2), patients received intravenous vancomycin but with different dosing regimens. In the prospective observational study of Blassmann et al., EVD-associated ventriculitis patients received a median daily dose of 2500 mg of vancomycin in the form of two prolonged infusions. Targeted serum trough concentrations were 10–15 mg/L. All patients received concomitant meropenem treatment; smaller percentages of patients received additional fosfomycin (33%) or dexamethasone. CSF penetration was poor, with a median CSF/serum ratio of only 3% and median AUC_0–24_ in serum and CSF of 455.09 mg∙h/L and 14.10 mg∙h/L, respectively. Very low vancomycin concentrations were found in 34% of CSF samples. After 30 days, no deaths were reported. A three-compartment linear population PK model suggested that the penetration of vancomycin into the CSF was even slower than the CSF clearance. Moreover, there was no correlation between vancomycin AUC in plasma and CSF. No covariates, such as CSF protein or CSF drain, influenced CSF penetration. Simulation of different dosing regimens showed that in cases of severe infections, for example, MRSA or methicillin-resistant *Staphylococcus epidermidis* (MRSE), where MIC ≥ 1 mg/L, a standard dosing regimen of 2000 mg every 12 h as a prolonged infusion most likely does not lead to sufficient CSF concentrations [36]. However, compared to an intermittent dosing regimen, the continuous infusion could be beneficial for reducing vancomycin-associated nephrotoxicity [46]; Consequently, TDM is recommended to improve patient therapy [36].

In the study of Jalusic et al., patients received a total daily dose of 2000–4000 mg vancomycin, depending on creatinine clearance, either as a continuous infusion or as an intermittent infusion every 6 h. Patients were treated additionally with ceftazidime. Targeted serum trough concentrations were 15–20 mg/L for bolus infusions and 20–25 mg/L for continuous infusion, aiming to achieve CSF trough levels of >1 mg/L. Median CSF penetration was higher in patients treated with bolus injections than in patients treated with continuous infusion. Interestingly, in both groups, the CSF penetration significantly correlated with markers of cerebral inflammation such as CSF lactate. In this patient population, median CSF lactate was 3.3 mmol/L, higher than the concentrations in healthy adults. Simulations of different dosing regimens revealed that dosing of 1350 mg/L every 8 h achieved target CSF concentrations above 1 mg/mL at a lactate concentration of 3.3 mmol/L, and 4000 mg every 24 h was recommended for continuous infusion. A linear three-compartmental model demonstrated that total vancomycin plasma clearance (Cl) depends on creatinine clearance and that CSF lactate concentration correlates with the clearance between the central and the CSF department. Therefore, Jalusic et al. recommend the application of TDM and consider the degree of inflammation, measured by CSF lactate, and renal function for vancomycin dosing [20].

In the study of Mader et al., patients received vancomycin and/or meropenem via continuous infusion. Patients were treated with an initial bolus of 30 mg/kg of vancomycin and subsequently received 30 mg/kg/day. The targeted serum concentration for vancomycin was 20–30 mg/L, which was set to attain a PD target of AUC/MIC above 400 for susceptible *Staphylococcus aureus* isolates. Out of all samples, 33% were below the target concentration. The targeted CSF concentration was 2 mg/L, which set to the breakpoint for susceptible *Staphylococcus aureus* isolates. With this regimen, the CSF penetration varied enormously between patients, ranging from 3% to 48%, and resulted in exceeding the targeted CSF concentration in only 70% of the cases. Death occurred in 32% of patients [11].

#### 2.2.2. Vancomycin in Meningitis

In all studies on the use of vancomycin in meningitis (Table 2), patients received vancomycin intravenously. Mounier et al. conducted a retrospective study on *S. epidermidis* healthcare-associated meningitis. Patients were treated via continuous infusion with a bolus infusion of 15 mg/kg followed by 60 mg/kg/day. Patients of this study showed a low inflammatory status, which was shown by low CSF protein and low serum C-reactive protein. Even though sufficient vancomycin serum concentrations were achieved, concentrations of vancomycin in CSF were not sufficient for an effective treatment. Therefore, the administration of vancomycin was replaced by other antibiotics, predominantly linezolid. Overall, Mounier et al. point out the high risk of treatment failure in patients without strong meningeal inflammation and suggest reassessing the use of vancomycin as a first-line treatment in this patient group [47].

In the study of Ishikawa et al., patients were treated with intermittent vancomycin infusions two to four times a day. Doses were adjusted using TDM to achieve serum trough concentrations of 15–20 mg/L. Two out of seven patients received CSF drainage of 200–300 mL per day. In two patients (with and without drainage), vancomycin treatment was ineffective. Ishikawa et al. found no significant correlation between serum and CSF concentration, and CSF/serum ratio was not significantly correlated with cell count or glucose levels in CSF. However, they found that the protein concentration in CSF/serum albumin ratio showed a strong positive correlation with the vancomycin CSF/serum ratio. Therefore, CSF/serum albumin ratio could help estimate CSF penetration in meningitis patients [48].

In the prospective study on patients with proven or highly suspected post-neurosurgical meningitis of Wang et al., patients received 500 mg vancomycin every 6 h. In patients with a negative culture of CSF, vancomycin was used in combination with ceftriaxone. A total of 54.5% of patients were categorized as cured, and the condition of the remaining 45.5% did improve after 3–5 days of treatment. Wang et al. investigated the connection between vancomycin CSF penetration and clinical outcome. In contrast to the previously described study, Wang et al. found a significant correlation of the vancomycin concentration in serum and CSF. Interestingly, no significant differences in clinical response were seen in patients with serum C_min_ over 15 mg/L (achieved in 9 of 22 patients) and under 15 mg/L. In addition, only CSF C_min_ was correlated with a positive clinical response, namely the decline of the number of white blood cells in the CSF. However, neither serum C_min_ nor CSF C_min_ or CSF penetration was associated with clinical response [49].

Taheri et al. conducted a randomized clinical trial with post-neurosurgical meningitis patients and compared intermittent and continuous infusion of vancomycin. All patients received an initial dose of 25 mg/kg over two hours followed by an infusion of 25 mg/kg every 12 h in the intermittent infusion group or by a continuous infusion of 50 mg/kg/day. In addition, patients of both groups were treated with meropenem. Dexamethasone was administered in the first two days. In both groups, serum and CSF vancomycin concentration were positively correlated. In the continuous infusion group, the mean CSF concentration was significantly higher than in patients who received intermittent infusion. CSF penetration, on the other hand, was not significantly different between the groups. Therapy was well-tolerated under both dosing regimens, and all patients recovered [50].

In another study, Cai et al. compared vancomycin penetration into CSF in community-acquired meningitis patients and postoperative intracranial infection patients. All patients received 1000 mg vancomycin every 12 h. Following the same initial vancomycin schedule, patients were given various antibiotics. CSF drainage was performed in all intracranial infection patients but in none of the meningitis patients. In patients with postoperative intracranial infections, vancomycin concentrations and CSF penetration were similar to meningitis patients. In addition, the average serum vancomycin was lower than the recommended concentrations (15–20 mg/L). Serum concentration was correlated to bodyweight but not age and serum creatinine. Serum and CSF concentrations were weakly correlated. In postoperative intracranial infection patients, CSF/serum ratio was correlated to white blood cell count in CSF, but this was not the case in meningitis patients. Furthermore, CSF protein and glucose level were not correlated with CSF/serum ratio in both patient groups [51].

Noguchi et al. presented the administration of intravenous vancomycin in a rare case of *Staphylococcus epidermidis* meningitis in a neutropenic patient that was unrelated to a neurosurgical device. As an empiric treatment, intravenous vancomycin and meropenem were administered. The central venous catheter was suspected to be the source of the patients’ bacteremia and was removed. After identification of *Staphylococcus epidermidis*, intravenous vancomycin treatment was continued with 1 g every 12 h, and rifampin was added orally. Noguchi et al. present this case as the first successfully treated patient with this specific disease condition [52].

In summary, the currently available data show the large variability of treatment outcomes of patients treated with vancomycin and that in many cases, the standard recommended dosing regimen might not be effective.

**Table 2:** Summary of vancomycins’ PK parameters in adults affected with ventriculitis and meningitis. *n* represents the number of participants.

Type of Infection	Study Design (*n*)	Dose	Route	Blood and CSF Sampling	Plasma (mg/L)	CSF (mg/L)	CSF Penetration	PK Model	Age	Treatment Outcome/Remarks	Ref.
Proven or suspected EVD- associated ventriculitis	Prospective observational (21)	Prolonged infusion (over 4 h) median daily dose 2500 (500–4000) mg in two divided doses, targeted trough concentrations in serum 10–15 mg/L	IV	Serum and CSF both just before start of infusion (C_min_) and at end of infusion (C_max_), Source of CSF samples: intraventricular (EVD)	C_max_ = 25.67 (10.60–50.78) C_min_ = 9.60 (4.46–23.56)	C_max_ = 0.65 (<0.24–3.83) C_min_ = 0.59 (<0.24–3.95)	Cumulative AUC_CSF_/CumulativeAUC_Serum_ 0.03 (0.01–0.18)	Yes	52 (46–80) years	30 days mortality: 0	[36]
EVD- associated ventriculitis	Retrospective (29)	Daily dose of 2–4 g, depending on creatinine clearance, either via continuous infusion (initial bolus of 1 g over 1 h) or as intermittent infusion (q6h over 1 h), depending on physician, Doses adjusted to TDM (target plasma trough levels of 15–20 mg/L with bolus infusions, plasma levels of 20–25 mg/L with continuous infusion)	IV	Mostly trough samples, no CSF samples for 3 patients, Source of CSF samples NS	17.7 (IQR 13.00, 23.02)	2.9 (IQR 1.76, 4.2)	0.13 (IQR 0.07; 0.24) under bolus therapy 0.08 (IQR 0.05; 0.12) under continuous therapy	Yes	52 (IQR 44; 61) years	NS	[20]
Ventriculitis	Retrospective(22 for vancomycin and meropenem)	Continuous infusion of 30 mg/kg/day after initial bolus of 30 mg/kg of adjusted body weight, Serum target concentration of 20–30 mg/L, CSF target concentration of 2 mg/L, Dosage adjusted according to TDM results	IV	Samples from 15 patients, timepoints NS, Source of CSF samples: NS	22 ± 814 values (33%) below and two values,(5%) above the targeted concentration	4.5 ± 2.6Above the breakpoint for susceptibility of *S. aureus* in 30 cases (70%), above the breakpoint for susceptibility of other Gram-positive cocci in 21 cases (49%)	20% ± 11% (3–48%)	No	57 ± 12 years	Death of 7 out of 22 patients, for the remaining patients GOS 2–4	[11]
Healthcare- associated meningitis	Retrospective (6)	15 mg/kg loading dose, followed by continuous infusion of 60 mg/kg/day	IV	First measurements (day 1–5 of treatment); when antibiotics administration discontinuous right before following administration Second measurements (day 2–11, 4 patients), Source of CSF samples: NS	36.1 ± 19.2 (A)(15.4–66.4) 34.9 ± 22.1 (A)(17.2–71.4)	3 patients < 1.1,2 patients 1.5 1 patient < 1.1 3 patients: 1.2, 2.6, 2.2	ND	No	43.2± 13.0 (28–64) years (A)	Treatment regimen was changed to other antibiotics	[47]
Suspected and provenbacterial meningitis	Retrospective (7)	2–4 times/day, Dose adjusted to TDMto achieve serum trough concentrations of 15–20 mg/L	IV	Blood samples measured just before vancomycin infusion when steady-state concentrations were achieved and after at least 2 days of the dosing regimen, CSF measured retrospectively using residual CSF, Source of CSF samples: intraventricular (EVD) or lumbar, after achievement of steady-state serum concentrations	17.6 ± 7.2	3.31 ± 3.14	0.180 ± 0.152 (0.010–0.431)	No	41.7 ± 19.2 (17–70) years (A)	Vancomycin treatment ineffective in 2 patients, for 2 patients clinical efficacy was undeterminable	[48]
Proven or highly suspectedpostsurgical meningitis	Prospective (22)	500 mg over 1 h, q6h(for at least 5 days)	IV	Serum and CSF both measured 5 h after the end of infusion (C_min_) on day 3 or 4, Source of CSF samples: lumbar (puncture or drainage)	C_min_ = 13.38 ± 5.36 (5.07–28.6)	C_min_ = 3.63 ± 1.64 (1.44–8.51)	0.291 ± 0.118 (0.163–0.570)	No	52.6 ± 12.1 (25–74) years	12 patients were cured, 10 patients improved after 3–5 days, no vancomycin-induced nephrotoxicity	[49]
Postneuro- surgical meningitis	Randomized clinical trial (20) (10 for each infusion group)	Intermittent infusion: Initial dose of 25 mg/kg over 2 h, then 25 mg/kg over 2 h q12h Continuous infusion: Initial dose of 25 mg/kg over 2 h, then 50 mg/kg/day	IV IV	Serum samples measured 30 min before (C_trough_) and 1 h after each maintenance dose (C_peak_), CSF samples measured at days 4 and 8, concomitantly with serum trough samples, Source of CSF samples: NS “-“	C_trough_ = 17.49 ± 2.46 C_peak_ = 41.33 ± 2.73 24.76 ± 2.02	C_trough_ = 4.83 ± 1.05 6.20 ± 1.31	CSF/trough ratio 27.39% ± 2.43% 24.84% ± 3.54%	No	49 ± 7.25 years 48 ± 8.02 years	Recovery of all patients, Therapy was well-tolerated	[50]
Community-acquired Meningitis Postoperative intracranial infection	Prospective (22) (10 community-acquired meningitis, 12 postoperative intracranial infection)	Initial treatment 1 g over more than 1 h, q12h; regimen adjusted according to signs and symptoms “-“	IV IV	Serum and CSF 0.5 h before fifth dose (C_trough_) “-“ Source of CSF samples: lumbar (puncture) or intraventricular (ventricle drainage tube)	C_trough_ = 9.81 ± 1.89 (6.90~13.00) C_trough_ = 9.74 ± 3.04 (5.01~13.90)	C_trough_ = 2.47 ± 1.15 (0.80~4.03) C_trough_ = 1.90 ± 1.29 (0.42~4.40)	0.26 ± 0.12 (0.11~0.47) 0.19 ± 0.12(0.06~0.45)	No	36.2 ±14.3 years 51.2 ± 9.9 years	NS	[51]
Meningitis	Case report	1 g, q12h	IV	Blood and serum samples measured during treatment, NS, Source of CSF samples: lumbar (puncture)	C_trough_ = 11–18 C_peak(day 26)_ = 28.6	C_trough_ = 9.4 C_(1 h after infusion)_ = 12.8	ND	No	47 years	Successfully treated	[52]

Abbreviations: C_max_: maximum concentration, C_min_: minimum concentration, C_peak_: peak concentration, C_trough_: trough concentration, GOS: Glasgow Outcome Scale, IQR: interquartile range, IV: intravenous, NS: not specified, ND: no data, q12h: every 12 h, TDM: therapeutic drug monitoring (A) not reported in study, calculated from individual patient data, “-“: same as above.

## 3. Meropenem

Meropenem is a broad-spectrum carbapenem antibiotic that is used in a broad range of serious infections, including CNS infections. It has activity against Gram-positive and Gram-negative pathogens and is especially important as empirical treatment of serious bacterial infections in hospitalized patients [53]. Meropenem is a time-dependent antibiotic, the relevant PD parameter of which is the percentage of the dosage interval in which the drug concentration remains above the MIC (%T > MIC) [54]. Recommended total daily dose of meropenem in healthcare-associated ventriculitis and meningitis is 120 mg/kg in infants and children and 6 g in adults, both in a dosing interval of 8 h [14].

### 3.1. Pediatrics

Recent studies reported on the use of intravenous meropenem in children (Table 3) included three studies on patients with meningitis and one case report of a ventriculitis patient. In the NeoMero studies, Germovsek et al. examined the PK of meropenem in infants younger than three months, for which meropenem is currently not licensed. Included were infants with suspected bacterial meningitis and infants with late-onset sepsis (LOS). Meningitis and LOS patients were treated intravenously with 40 mg/kg and 20 mg/kg meropenem, respectively, with a treatment interval of 8 or 12 h, depending on the age of the patients. Plasma and CSF concentrations in meningitis patients showed very high interindividual variability (Table 3). Data of both patient groups were used for a one-compartmental PK model, which showed that serum creatinine clearance significantly affects meropenem clearance. In addition, CSF lactate concentration and CSF total protein concentration were connected to CSF penetration of meropenem. The estimated meropenem penetration into the CSF was 8.4%. However, in patients with inflamed meninges indicated by increased CSF protein concentration, penetration was predicted to reach over 40% at a protein concentration above 6 g/L. Simulations of bolus and continuous infusion of 20 mg/kg and 40 mg/kg dosing demonstrated that continuous infusion increases plasma %T > MIC but decreases %T > MIC in CSF [55].

Ohata et al. used data from pediatric patients with bacterial meningitis and other infections for creating a three-compartment population PK model for meropenem infusion. The majority of included patients received 40 mg/kg meropenem every 8 h. All children with bacterial meningitis received dexamethasone concomitantly for the first 4 days of therapy. The overall mortality rate was low with this therapeutic approach, with 3 deaths reported out of 117 meningitis patients. For creating the PK model of meropenem in CSF, samples from five adults with bacterial meningitis, who received 2 g meropenem every 8 h, were also included. Simulations of different meropenem dosages (20, 40, 80 mg/kg; every 8 h) and infusion durations (0.5, 2, 4 h) demonstrated that an increase in dosing leads to a distinct increase in target attainment rates (50%T > MIC in CSF) but that increased infusion duration is advantageous only for target attainment in plasma and not in CSF. Thirty-six pediatric patients, for which pathogen-specific MICs were available, had a satisfactory clinical response and showed at least 75.3%T > MIC in CSF. CSF penetration of meropenem (population mean CSF/plasma AUC ratio) was estimated as 0.146. In patients with bacterial meningitis younger than 6 months, meropenem AUC in CSF and the CSF/plasma AUC ratio were higher than in older pediatric patients. In addition, patients with glucose ratios under the threshold for bacterial meningitis (<0.4) had a significantly greater CSF/plasma AUC ratio. Based on their results, Ohata et al. suggest a dosing regimen of 40 mg/kg every 8 h for pediatric patients with bacterial meningitis. However, it was estimated that with pathogens with MIC > 1 mg/L, sufficient meropenem concentrations could only be achieved in less than half of this patient population [56].

Verscheijden et al. developed a physiologically based PK model (PBPK) for pediatric patients that can be used for meropenem in bacterial meningitis patients. Simulated AUC CSF/AUC plasma ratios ranged from 0.09 to 0.12. Overall, in comparison to relatively healthy individuals, patients with sepsis or meningitis had higher AUC CSF/AUC_serum_ ratios [57].

Cies et al. reported the case of a 2-year-old patient diagnosed with *Serratia marcescens* ventriculitis, in which changing the antibacterial treatment from intermittent to continuous intravenous infusion of meropenem led to a successful clinical outcome. After unsuccessful treatment with cefotaxime, meropenem was administered at a 40 mg/kg dosing regimen every 6 h, and amikacin was used additionally. Due to remaining positive CSF cultures, treatment was changed to continuous 200 mg/kg/day infusion. With the PD target set to ≥40% free time above MIC (≥40% *f*T > MIC), the continuous infusion of meropenem led to a probability of target attainment of 100% in serum and CSF. The patient had a computed meropenem clearance from the serum of 10.2 mL/kg/min, which is significantly higher than the population PK estimates generated from both healthy volunteers and other pediatric ICU patients. Cies et al. highlight the importance of TDM in serum and CSF to ensure sufficient drug exposure [58].

### 3.2. Adults

#### 3.2.1. Meropenem in Ventriculitis

Only two studies, which use an intermittent and continuous dosing approach, are published on intravenous meropenem treatment in ventriculitis (Table 4).

Blassmann et al. reported a prospective observational study in which meropenem was given as a prolonged 4 h infusion at a dose of 1000 mg every 8 h for patients with adverse drug effects or renal impairment and at a dose of 2000 mg every 8 h for the remaining patients. In addition to meropenem, all patients received vancomycin, which was replaced by linezolid in one patient. Some patients concomitantly received dexamethasone or fosfomycin. The median AUC_0–24_ in serum and CSF were 350.22 mg∙h/L and 26.56 mg∙h/L, respectively. No deaths were reported after 30 days. A three-compartment linear population PK model was developed, which identified no covariates. Simulation of different dosing regimens (2000–5000 mg, every 8 or 6 h) showed that CSF trough concentration of 2 mg/L could only be exceeded in 53.8% of simulated patients when treated with 2000 mg meropenem every 8 h but exceeded 95.1% in simulated patients that received 5000 mg every 6 h. Due to the high interindividual variability in reported concentrations and CSF/serum ratios, Blassmann et al. recommend TDM in serum and CSF [35].

Mader et al. conducted a retrospective analysis on continuous infusion of meropenem in ventriculitis patients. Patients received an initial bolus of 1000 mg meropenem, followed by administration of 6000 mg/day. Dosages were adjusted to TDM results. Serum concentrations were aimed to be between 16 and 32 mg/L, representing 8–16 times the breakpoint of susceptibility for Gram-negative rods such as *Pseudomonas aeruginosa*, and CSF concentrations were kept above 2 mg/L. The mean penetration of meropenem showed high interindividual differences (Table 4). The meropenem concentrations in CSF exceeded the breakpoint for susceptibility for Gram-negative rods in 78% of cases. Mader et al. state that for adult ventriculitis patients, routine TDM is beneficial and feasible to ensure efficient CSF concentrations for continuous infusion of meropenem [11].

#### 3.2.2. Meropenem in Meningitis

Only two studies are reported in the use of meropenem in adults affected with meningitis. In the first study, Zhang et al. conducted a prospective open-label study to examine the PK of meropenem in patients with post-neurosurgical meningitis (Table 4). Patients were treated intravenously with three different dosing regimens, depending on disease severity (1 g every 8 h, 1 g every 6 h, or 2 g every 8 h). In most patients, meropenem treatment was combined with vancomycin or norvancomycin. In the two groups with higher daily total dose, penetration of meropenem into CSF was higher. However, since the dosage regimen was adjusted to disease severity, Zhang et al. point out that meropenem penetration presumably is related to the severity of meningitis. All treatment regimens led to favorable treatment responses in most patients. For patients treated with 1 g meropenem every 8 or 6 h, this was the case for 88.1% and 94.7%, respectively, while treatment with 2 g every 8 h led to 76.1%. Only one adverse event was identified as possibly related to the meropenem treatment, which was a case of skin rash. All dosing regimens led to a %fT > MIC in plasma of at least 40% for both *Enterobacteriaceae* (MIC = 1 mg/L) and *Acinetobacter baumannii* (MIC = 2 mg/L) caused meningitis. Overall, the results indicated that higher dose and shorter dosing intervals (2 g every 8 h and 1 g every 6 h) are more efficient for the treatment of this patient population [59].

Lu et al. developed a two-compartment model with an additional CSF compartment for intravenous meropenem in patients with bacterial meningitis after neurosurgery and especially paid attention to the influence of CSF drainage volume. The model identified no significant covariate. Monte Carlo simulation was performed, and PD targets were chosen as 40%T > MIC in plasma and 50% and 100% T > MIC in CSF. Simulations showed a higher probability of target attainment with prolonged infusions and that increased CSF drainage results in a lower probability of target attainment in CSF. Based on their results, Lu et al. recommend a meropenem dosing regimen of 2 g every 8 h as a 4 h infusion and a CSF drainage rate of fewer than 150 mL/day for the highest probability of target attainment. With this dosing regimen, there was a >80% probability of attaining T > MIC in CSF of 50% and 100% for MICs ≤1 and ≤0.5 mg/L, respectively [22].

##### Main Highlights

Based on the aforementioned data, both vancomycin and meropenem were administered as an intermittent or continuous infusion, irrespective of the type of infection. However, based on the reviewed papers, which dosing regimen resulted in a better clinical outcome cannot be clearly stated. For instance, for meropenem, a simulation of intermittent and continuous infusion showed that the latter one leads to lower %T > MIC in CSF in infants; however, the switch from intermittent to continuous infusion enabled successful treatment in a pediatric patient. Interestingly, conflicting data exist for vancomycin, whereby direct comparison of continuous and intermittent administration in a study with meningitis patients showed higher CSF concentrations under continuous infusion but no difference in CSF penetration. In contrast, in another study on ventriculitis patients, CSF penetration was slightly higher with intermittent vancomycin infusion.

Interestingly, in 3 out of 10 papers, the authors report that intravenous administration of vancomycin was ineffective for at least part of the patients. However, it is not possible to rate the overall success of the intravenous vancomycin treatment since it was administered in combination with other antibiotics in many of the discussed cases. It is worth mentioning that intraventricular administration of antibiotics, which was only used in the case of vancomycin in infants with ventriculitis, is not connected to adverse events, and higher CSF concentrations can be achieved. Therefore, the intraventricular administration could be beneficial for treating pathogens with high MICs; yet, it needs to be further elucidated, in which patient groups intraventricular administration of vancomycin is more beneficial or necessary to improve treatment outcomes.

Finally, when comparing the PK parameters and available treatment outcomes of all reviewed studies, no superior dosing regimen for vancomycin or meropenem could be identified, neither for meningitis nor ventriculitis.

## 4. Conclusions and Future Perspectives

This review presents PK data on the use of meropenem and vancomycin in pediatric and adult patients with ventriculitis and meningitis. Intraventricular administration of vancomycin was only practiced in two studies, both of them in infant ventriculitis, while in the vast majority of studies, meropenem and vancomycin were administered intravenously as an intermittent or continuous infusion in adult meningitis and ventriculitis patients. Interestingly, the dosing regimens varied between studies despite treating patients with the same type of infection (meningitis or ventriculitis). In many of the included studies, concentrations of vancomycin and meropenem in serum and CSF showed high interindividual variability, which also applied to the penetration of both antibiotics into the CSF. Eight out of the 18 evaluated publications contained a PK model. For vancomycin, all PK models were regarding its use in ventriculitis. According to the simulations of different treatment regimens, one study recommended the adaption of dosing to the degree of inflammation and renal function, while another study recommends further investigation of continuous infusion for reduction in renal toxicity. For treatment of bacterial meningitis, CSF/serum albumin ratio was identified as a potential predictor of CSF penetration of vancomycin, while a continuous infusion of vancomycin showed no impact on CSF penetration in this patient population. Five out of the eight publications on meropenem included a PK model. Overall, six studies specifically recommend therapeutic drug monitoring of serum and CSF to ensure efficient concentrations. Due to high variability in the dosing regimen and high inter- and intra-individual variability outcome of treating meningitis and ventriculitis, larger studies are necessary to identify the optimal dosing regimens that are well-suited not only for the type of infection but also for the individual patient. In pediatric patients, four out of the seven available studies on the use of meropenem and vancomycin contain a PK model. The obtained data demonstrate that PK modeling in this patient population is equally, if not more substantial, than in adults. Overall, parameters that could potentially impact the PK properties of vancomycin and meropenem in ventriculitis and meningitis patients are still controversial. Finally, further characterization could help achieve better target attainment and treatment outcomes.

## Figures and Tables

**Table 3:** Summary of meropenem’s PK parameters in pediatrics affected with meningitis and other infections. *n* represents the number of participants.

Type of Infection	Study Design (*n*)	Dose	Route	Blood and CSF Sampling	Plasma (mg/L)	CSF (mg/L)	CSF Penetration	PK Model	Age	Treatment Outcome/ Remarks	Ref.
Meningitis Late-onset sepsis	Prospective (49) (123) (167) Combined	40 mg/kg, q12h in patients with <32 weeks GA and <2 weeks PNA, q8h in all other patients, infused over 30 min 20 mg/kg, q12h in patients with <32 weeks GA and <2 weeks PNA, q8h in all other patients, infused over 30 min	IV IV	Plasma samples: immediately at the end of infusion, 5–6 h post-dose for q8h or 7–8 h post-dose for q12h, immediately before dosing in majority of patients, or only trough samples CSF samples measured opportunistically 5.27 (0–12.0) h post-dose in 56 patients, Source CSF samples: lumbar (puncture) “-“	12.4 (0.1–139.0) 5.27 (0.01–147.7) 7.94 (0.01–147.7)	1.90 (0.05–35.4) 1.23 (0.04–7.34) 1.58 (0.04–35.4)	ND ND model-based typical estimate 8.4%	Yes	GA: 37.1 (23.4–41.9) weeks PNA: 9 (1–90) days; PMA: 38.8 (24.9–51.1) weeks GA: 31.9 (22.6–41.3) Weeks PNA: 15 (3–83) days PMA: 36.0 (23.7–51.3) weeks GA: 33.3 (22.6–41.9) Weeks PNA: 13 (1–90) days PMA: 37.4 (23.7– 51.3) weeks	NS discussed for 24 LOS patients, 12 patients successfully treated with meropenem	[55]
Meningitis and otherinfections	Meta-Analysis of three clinical studies (154 children,5 adults included)	Children: 10, 20 or 40 mg/kg q8h, depending on study and disease severity, infused over ≥0.5 h Adults: 2 g, q8h, over ≥0.5 h	IV	Serum samples from patients with various infections, CSF samples from patients with bacterial meningitis, Blood and CSF samples collected after more than three doses. During or after completion of infusion (up to 6.5 h), Source of CSF samples: lumbar (puncture)	28.7 ± 29.1	1.82 ± 2.7	Estimated population mean CSF/plasma AUC ratio 0.146	Yes	30.6 ± 34.4 months, 60.6 ± 15.9 years	Clinical outcomes reported for 117 bacterial meningitis patients: 58 patients cured without sequelae, 56 with mild or severe sequelae, and 3 reported deaths	[56]
Sepsis/ bacterial meningitis	PK study						Simulated AUC_CSF_/AUC _Serum_ 0.09–0.12	Yes		NS	[57]
EVD- related ventriculitis	Case report	40 mg/kg over 0.5 h, q6h Hospital day 27: 200 mg/kg/day	IV	Serum and CSF measured simultaneously 2 and 4 h after infusion, Source of CSF samples: NS NS	C_(2 h)_ = 12 C_(4 h)_ = UD C_(day 33)_ = 13 C_(day 37)_ = 15	C_(2 h)_ = 1 C_(4 h)_ = 0.5 C_(day 33)_ = 0.5 C_(day 37)_ = 0.5	3%	No	2 years	Successfully treated	[58]

Abbreviations: AUC: area under the curve, GA: gastrointestinal age, IV: intravenous, NS: not specified, ND: no data, PMA: postmenstrual age, PNA: post-natal age, q12h: every 12 h, UD: undetectable, LOS: late-onset sepsis, “-“: same as above.

**Table 4:** Summary of meropenem’ PK parameters in adults affected with ventriculitis and meningitis. N represents the number of participants.

Type of Infection	Study Design (N)	Dose	Route	Blood and CSF Sampling	Plasma (mg/L)	CSF (mg/L)	CSF Penetration	PK Model	Age	Treatment Outcome/ Remarks	Ref.
Proven or suspected ventriculitis	Prospective observational (21)	1 g q8h (adverse drug effects/renal impairment) or 2 g q8h, both over 4 h	IV	Plasma and CSF samples measured just before the start of infusion and after the end of infusion, Source of CSF samples: intraventricular (intraventricular catheter)	C_min_ = 2.54 (0.00–31.40) C_max_ = 20.16 (4.40–69.00)	C_trough_ = 1.28 (0.00–4.10) C_(after 4 h)_ = 1.20 (0.00–6.20)	Cumulative AUC_CSF_/ CumulativeAUC_Serum_ 0.09 (0.03–0.16)	Yes	52 (46–80) years	30 days mortality: 0	[35]
Ventriculitis	Retrospective (22, for both vancomycin and meropenem)	Continuous infusion 6 g/day after initial bolus of 1 g over 30 min Serum target concentration of 16–32 mg/L CSF target concentration of 2 mg/L Dosages adjusted according to TDM results	IV	Samples from 20 patients, timepoints NS, Source of CSF samples: NS	30.7 ± 14.9 mg/L two values (6%) below and nine values (25%) above target concentration	5.5 ± 5.2 mg/L above the break- point of susceptibility for Gram-negative rods in 24 cases (78%)	18% ± 12% (2–40%)	No	57 ± 12 years	Death of 7 out of 22 patients, the remaining patients GOS 2–4	[11]
Post-neurosurgical meningitis	Prospective (82)	2 g q8h, 1 g q8h, or 1 g q6h depending on their baseline conditions Infusion rate 1 g/h	IV	Blood and CSF samples collected simultaneously after the fourth meropenem dose at different time points ranging from during the infusion to immediately before administration of next dose, Source of CSF samples: lumbar (drainage) or intraventricular (EVD)	2 g q8h: C_peak_ = 43.2 ± 5.3 1 g q8h: C_peak_ = 28.9 ± 2.7 1 g q6h: C_peak_ = 31.5 ± 3.4	2 g q8h: C_peak_ = 2.4 ± 0.3 1 g q8h: C_peak_ = 1.2 ± 0.2 1 g q6h: C_peak_ = 1.6 ± 0.2	2 g q8h: P_max_ = 17.6% ± 7.3% 1 g q8h: P_max_ = 14.3% ± 1.7% 1 g q6h: P_max_ = 30.9% ± 24.2%	Yes [22]	43.4 ±13.1 (19–77) years	2 g q8h: favorable treatment response in 76.1% of patients, 1 g q8h: 88.1%, 1 g q6h: 94.7%, One possibly related adverse event in one patient (skin rash)	[59]

Abbreviations: C_max_: maximum concentration, C_min_: minimum concentration, C_Peak_: peak concentration, C_trough_: trough concentration, IV: intravenous, NS: not specified, P_max_: maximal percent penetration (= AUC_0–∞_ (CSF)/AUC_0–∞_ (plasma) × 100%), TDM: therapeutic drug monitoring.

## Data Availability

Not applicable.
